# Genome-wide analysis of poplar NF-YB gene family and identified *PtNF-YB1* important in regulate flowering timing in transgenic plants

**DOI:** 10.1186/s12870-019-1863-2

**Published:** 2019-06-11

**Authors:** Rongkai Wang, Ling Zhu, Yi Zhang, Junfeng Fan, Lingli Li

**Affiliations:** College of Forestry, Northwest A&F University, Yangling, 712100 China

**Keywords:** Poplar, Genome-wide analysis, *PtNF-YB1*, Flowering time, Transgenic plant

## Abstract

**Background:**

Compared with annual herbaceous plants, woody perennials require a longer period of juvenile phase to flowering, and many traits can be only expressed in adulthood, which seriously makes the breeding efficiency of new varieties slower. For the study of poplar early flowering, the main focus is on the study *Arabidopsis* homologue gene *CO*/*FT*. Based on studies of *Arabidopsis*, rice and other plant species, some important research progress has been made on the regulation of flowering time by NF-Y subunits. However, little is known about the function of NF-Y regulating flowering in poplar.

**Results:**

In the present study, we have identified *PtNF-YB* family members in poplar and focus on the function of the *PtNF-YB1* regulate flowering timing using transgenic *Arabidopsis* and tomato. To understand this mechanisms, the expression levels of three known flowering genes (*CO*, *FT* and *SOC1*) were examined with RT-PCR in transgenic *Arabidopsis*. We used the Y2H and BiFC to assay the interactions between PtNF-YB1 and PtCO (PtCO1 and PtCO2) proteins. Finally, the potential molecular mechanism model in which *PtNF-YB1* play a role in regulating flowering in poplar was discussed.

**Conclusions:**

In this study, we have characterized the poplar *NF-YB* gene family and confirmed the function of the *PtNF-YB1* regulate flowering timing. At the same time, we found that the function of *PtNF-YB1* to improve early flowering can overcome species barriers. Therefore, *PtNF-YB1* can be used as a potential candidate gene to improve early flowering by genetic transformation in poplar and other crops.

**Electronic supplementary material:**

The online version of this article (10.1186/s12870-019-1863-2) contains supplementary material, which is available to authorized users.

## Background

Compared with annual herbaceous plants, woody perennials plants need longer juvenile phase to enter the flowering stage, and many traits can only be expressed in adulthood, which will seriously affect the breeding efficiency of woody plants. In poplar, for example, the juvenile phase generally lasts from 7 to 10 years, then trees begin flowering [1–4]. Before reaching the reproductive growth periods, selection efficiency is limited, since plant materials with genetic development relationships cannot be provided for breeding aiming at improving efficiency, quality and robustness.

Promoting early flowering of trees and shortening their juvenile phase can effectively shorten the traditional cross breeding cycle, accelerate the breeding process, and increase the breeding efficiency [5]. Therefore, the research on the mechanism of early-flower induction is not only the need to promote the development of forestry science, but also the key to understanding the molecular mechanism of sexual reproduction in plants. However, little is known about the physiological and genetic factors involving the flower induction in poplar.

The NF-Y (Nuclear Factor Y) transcription factor is a trans-acting factor that binds to the CCAAT box upstream of the promoter of a gene to regulate gene transcription and is present in almost all eukaryotic genomes, regulating the expression of many genes [6–8]. In mammals and plants, NF-Y is a heterotrimer composed of three subunits: NF-YA (HAP2/CBF-A), NF-YB (HAP3/CBF-B), and NF-YC (HAP5/CBF-C), which are required for the formation of NF-Y-DNA complex; while, the complex includes four subunits: HAP2, HAP3, HAP4, and HAP5 in yeast [9]. In yeast and mammals, each NF-Y subunit is encoded by a single gene; but in plants, it is encoded by multiple genes, and the number of genes encoding individual subunit is also different with species. For instance, in *Arabidopsis*, there are 10 genes encoding NF-YA subunits, 13 genes encoding NF-YB subunits, and 13 genes encoding NF-YC subunits. But in rice, the genes encoding each NF-Y subunit are 10, 11 and 7, respectively [10]. Relative to the detailed and extensive studies of the function of NF-Y subunits and their complexes in yeast and mammals, little is known about their biological function in plants.

Studies in recent years have shown that individual NF-Y subunits in plants are involved in many important growth processes, especially in embryogenesis [11, 12] and seed maturation [13–15], chloroplast synthesis [16–18], tissue division [19] and others processes. Simultaneously, the NF-Y subunit also plays an important role in response to stress, such as drought stress [20–25]. It is worth noting that more and more studies have found that the NF-Y subunit participates in the photoperiodic regulation of flowering induction pathways, and that different subunits function differently [26–34]. For example, Cai et al. found that the *AtNF-YB2* promotes the flowering process by increasing expression of the flowering key genes FLOWERING LOCUS T (FT) and SUPPRESSOR OF OVER EXPRESSION OF CONSTANS1 (SOC1) [27]. Concurrently, AtNF-YB2 and AtNF-YB3 can interact with At-NF-YC3, 4, 9, which play important roles in the control of flowering time via the photoperiod pathway [33]. In addition, Hackenberg et al. demonstrated that *AtNF-YC1* and *AtNF-YC2* over-expression induce early flowering, and the transcript levels of *FT* genes in plants were significantly increased [30]. Interestingly, the regulation of flowering time by NF-Y in rice is exactly the opposite of *Arabidopsis*. Transcription factor *NF-YB11* negatively regulates the flowering time by down-regulating the expression of flowering-related genes [35–37]. This also shows that the regulation mechanism of NF-Y in flowering time varies in different species.

Based on studies of *Arabidopsis*, rice and other plant species, some important research progress has been made on the regulation of flowering time by NF-Y subunits, and to unveiling the molecular mechanism. However, little is known about the function of NF-Y regulating flowering in poplar. In this study, we have characterized the poplar *NF-YB* gene family and confirmed the function of the *NF-YB1* (*PtNF-YB1*) regulate flowering timing using transgenic *Arabidopsis* and tomato. Finally, the potential molecular mechanism model of *PtNF-YB1* involved in flowering regulation was discussed.

## Results and discussion

### Identification of poplar PtNF-YBs

In order to identify poplar analogs of PtNF-YB proteins, amino acid sequences of *Arabidopsis* and rice NF-YBs sequences were used to search against the Phytozome database *Populus trichocarpa* V3.0 (https://phytozome.jgi.doe.gov/pz/portal.html). According to nomenclature of NF-YBs in *Arabidopsis* and rice, the genes were named as follows (Table 1).

**Table 1:**
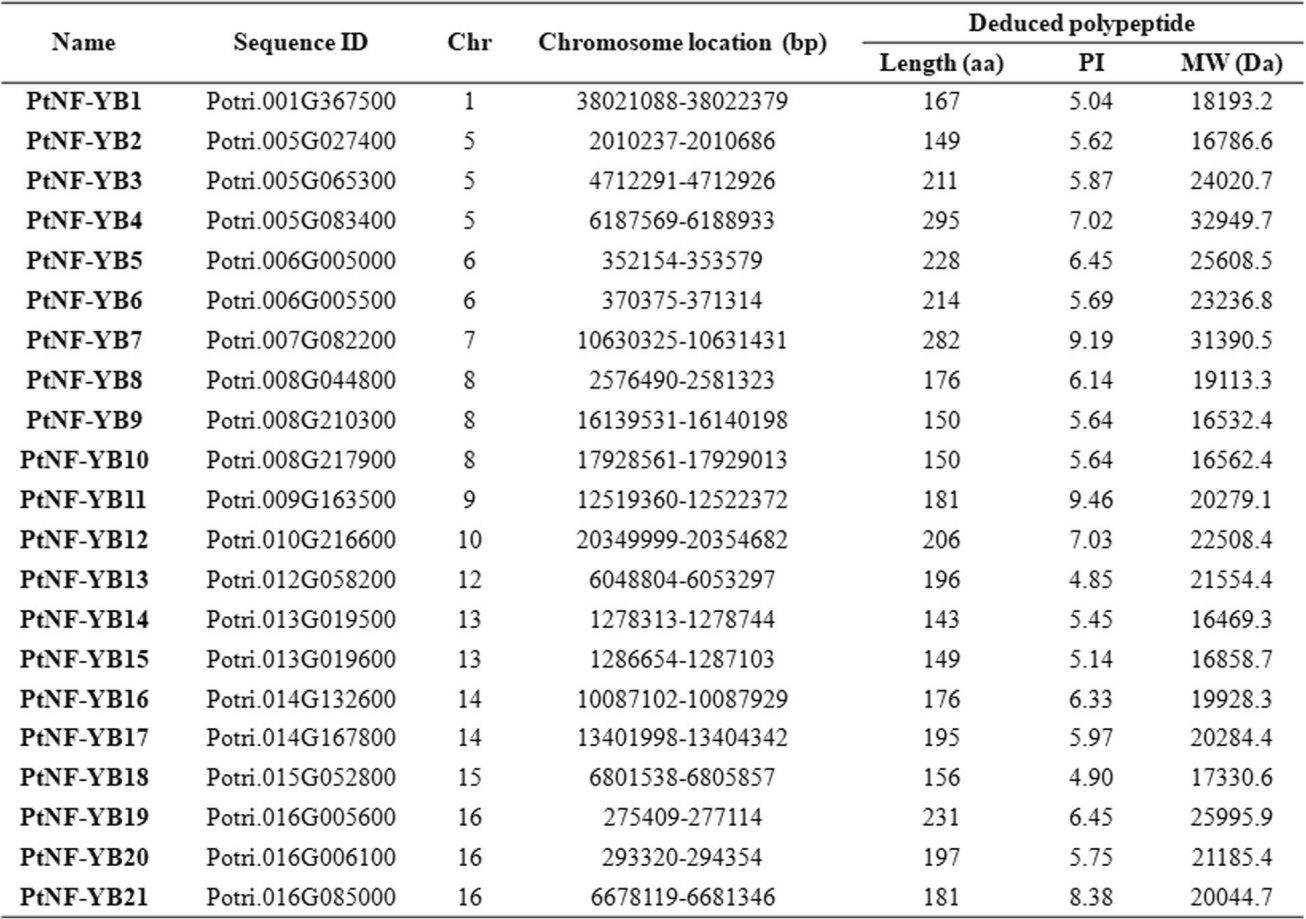
NF-YB transcription factors in poplar

The identified *PtNF-YB* genes in poplar encode proteins ranging from 143 to 295 amino acids in length with an average of 192 amino acids. The detailed information of *PtNF-YB* family genes in poplar, including sequence ID, chromosome location, amino acid length (aa), protein isoelectric point (PI) value and protein molecular weight (MW) (Da) was listed in Table 1.

To study the phylogenetic relationship between NF-YBs proteins in poplars, we constructed a unrooted tree based on the alignment of the NF-YBs full-length protein sequences (Additional file 1a). The phylogenetic tree was constructed using MEGA V5.5 by employing the Neighbor-Joining (NJ). As showed in the phylogenetic tree, it divided the PtNF-YBs family proteins into two distinct subgroups.

To better understand the functional prediction of PtNF-YBs, 10 conserved motifs were identifed using MEME V4.12.0 (Additional file 1b). As expected, we found that most of the closely related members of the phylogenetic tree share a common motif composition, indicating that there is a clear functional similarity between the NF-YBs proteins in the same subfamily.

### Analysis of the deduced amino acid sequence of PtNF-YB1

**Fig. 1:**
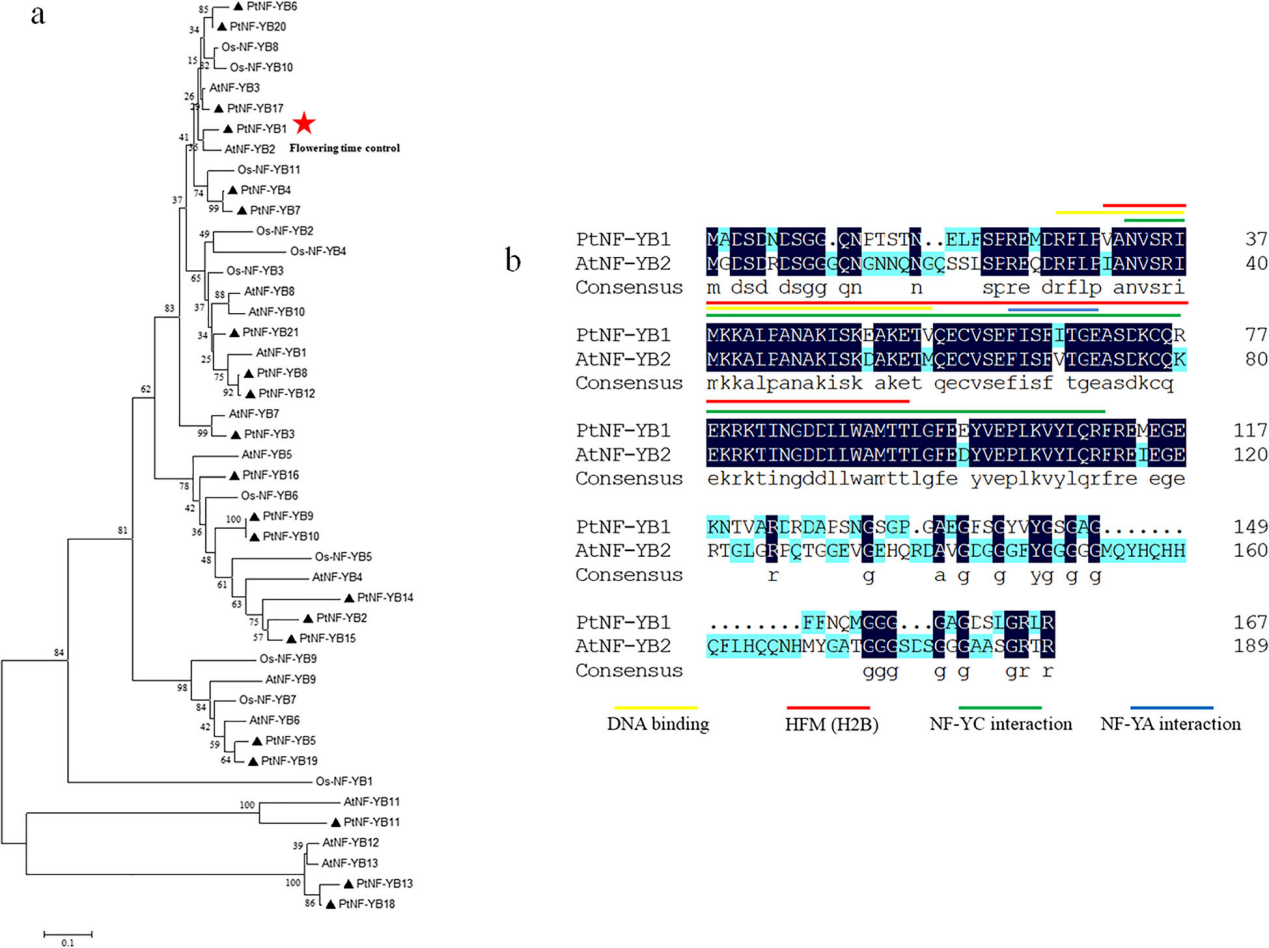
Analysis of the deduced amino acid sequence of PtNF-YB1. **a** Phylogenetic tree of poplar, *Arabidopsis* and rice. The phylogenetic trees were constructed by the Neighbor-Joining (NJ) method using conserved and amino acid sequences (MEGA V5.5). **b** Multiple sequences alignment of the conversed domains between PtNF-YB1 and AtNF-YB2

To investigate the evolutionary relationship, a phylogenetic analysis was made using the deduced amino acid sequence from poplar, *Arabidopsis* and rice based on the coding sequences of 21 PtNF-YBs, 13 AtNF-YBs and 11 OsNF-YBs (Fig. 1a). When compared with *Arabidopsis* and rice NF-YBs, it showed that *PtNF-YB1* formed a close cluster with *AtNF-YB2*, and it has been associated with flowering time [27, 38] (Fig. 1a). The *PtNF-YB1* gene encoded a predicted polypeptide with 167 amino acid residues, the protein molecular weight (MW) is 18,193.2 Da and the protein isoelectric point (PI) value is 5.04 (Table 1). Just like *Arabidopsis* AtNF-YB2, amino acid sequence alignment showed that the poplar PtNF-YB1 contained the DNA binding domain, the NF-YC interaction and the NF-YA interaction domain [39–41]. The histone-fold motif (HFM) of the core histone H2B was also observed in PtNF-YB1 [42] (Fig. 1b).

### Temporal and spatial expression patterns of *PtNF-YB1* gene

**Fig. 2:**
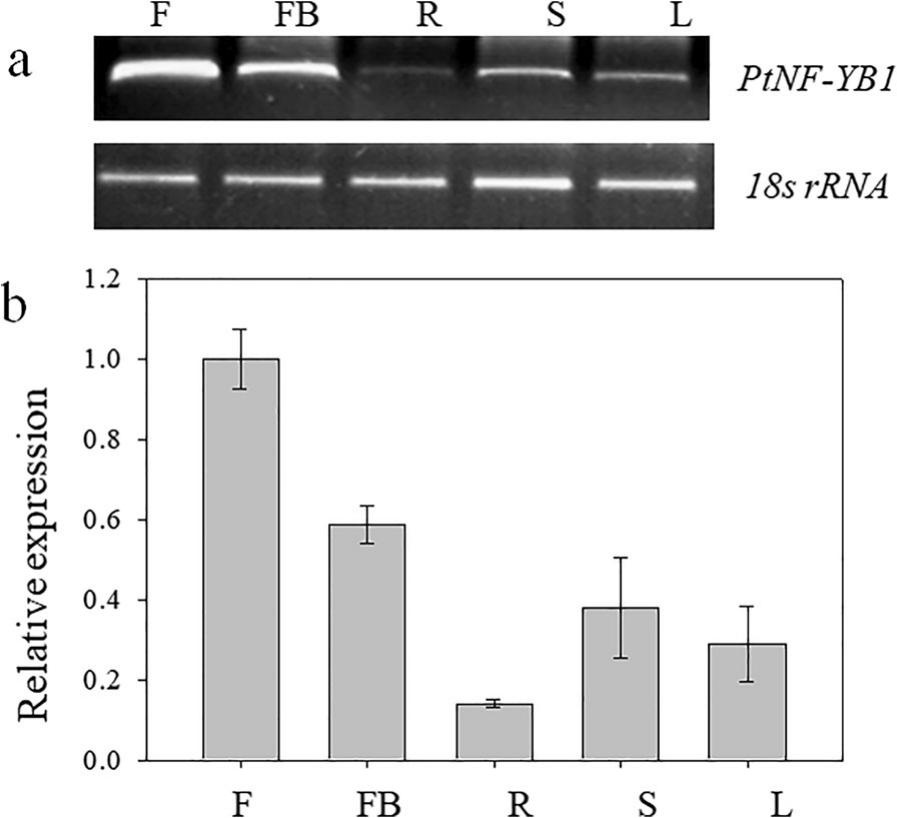
The temporal and spatial expression patterns of *PtNF-YB1*. **a** Expression analysis of *PtNF-YB1* gene with semi-quantitative RT-RCRs. **b** Expression analysis of *PtNF-YB1* gene with qRT–PCR. The five types of tissues were flowering (F), foral buds (FB), root (R), stem (S) and leaf (L). The RT-PCR reactions were repeated three times

To identify temporal and spatial expression patterns of *PtNF-YB1* gene, semi-quantitative RT-PCRs were conducted*.* The results indicated that *PtNF-YB1* was expressed in all five types of tissues: flowering (F), foral buds (FB), root (R), stem (S) and leaf (L). Among the five types of tissues, the flowering (F) and foral buds (FB) generated the higher level *PtNF-YB1* transcripts, the root (R) generated the lowest level (Fig. 2a). The qRT–PCR was also performed to confirm the results. The results showed the similar trends with the semi-quantitative RT-PCRs (Fig. 2b). It suggesting that *PtNF-YB1* may be part of the regulation of flowering pathway, just like *AtNF-YB2* [25, 39].

### Ectopic expression of *PtNF-YB1* improves early flowering in transgenic *Arabidopsis*

**Fig. 3:**
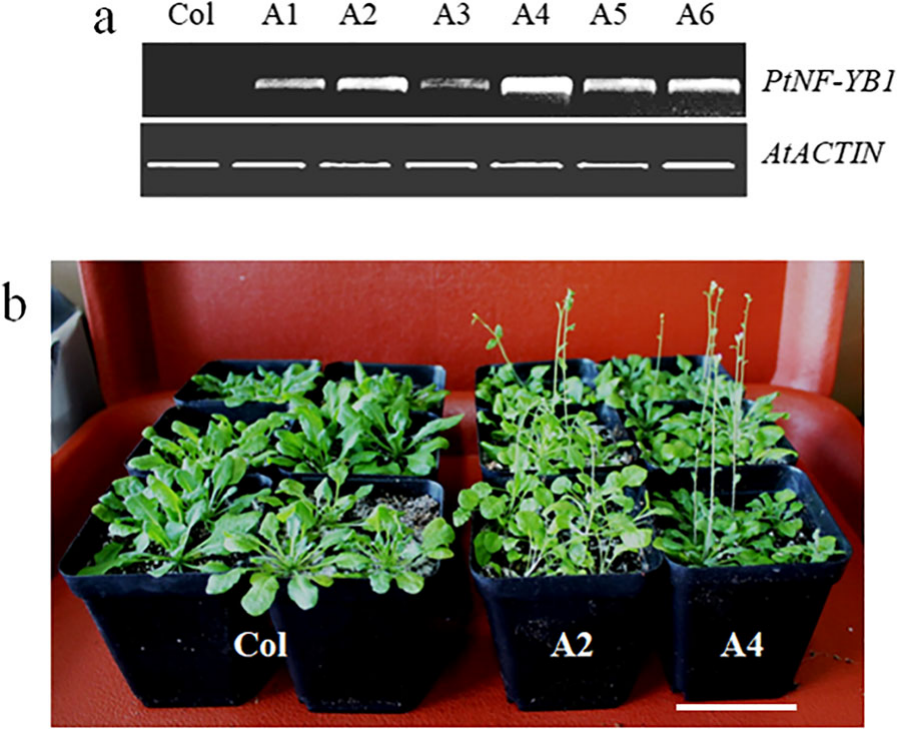
Phenotypes of transgenic *Arabidopsis* with *PtNF-YB1* gene under long day (LD) conditions. **a** Expression analysis of *PtNF-YB1* gene with semi-quantitative RT-RCRs in transgenic *Arabidopsis* lines. **b** Appearance of wild-type (Col) and transgenic *Arabidopsis* lines (A2 and A4) 28 days after transfer to the growth chamber. Bar = 6 cm

To determine the effects of poplar *PtNF-YB1* gene on flowering time, we generated *PtNF-YB1* over-expressing transgenic *Arabidopsis* plants. Consequently, more than 10 independent transgenic lines were obtained. Among them, 6 independent lines were used for further analysis (Fig. 3a). The T2 generation per line ware grown in the long day conditions (LD, 16 h light/8 h dark) and their phenotypes were examined. The transgenic *Arabidopsis* lines (A2 and A4) were flowered significantly earlier than the wild-type (Col) (Fig. 3b, Additional file 2). For example, *PtNF-YB1* transgenic line-A4 flowered with 7.1 rosette leaves and 3.4 cauline leaves, while wild-type (Col) flowered with 12.5 rosette leaves and 5.1 cauline leaves (Additional file 2). The result showed that *PtNF-YB1* ectopic expression noticeably improves early flowering in transgenic *Arabidopsis*.

### *PtNF-YB1* ectopic expression verifies its functions in promote early flowering in tomato

**Fig. 4:**
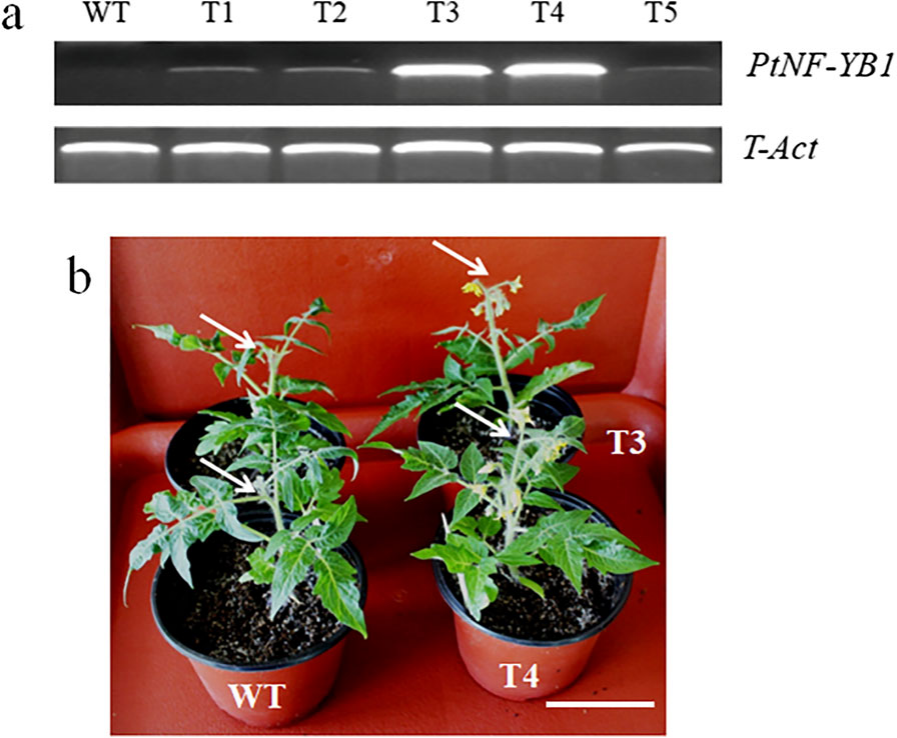
Over-expressing *PtNF-YB1* improves early flowering in transgenic tomato. **a** Expression analysis of *PtNF-YB1* gene with semi-quantitative RT-RCRs in transgenic *Arabidopsis* lines. **b** The wild-type (WT) and transgenic tomato lines (T3 and T4) 55 days after transfer to the growth chamber. Bar = 10 cm

To verifies *PtNF-YB1* functions in promote early flowering, we also generated *PtNF-YB1*-overexpressing transgenic tomato. We obtained 8 independent transgenic lines and 5 independent lines were used for further analysis (Fig. 4a). The T2 generation were grown in the nursery soils pots and the greenhouse conditions at day (25 °C) and night (20 °C). The *PtNF-YB1*-overexpressing tomato lines (T3 and T4) were also flowered significantly earlier than wild-type (WT) plants under same growth environment (Fig. 4b). For example, *PtNF-YB1* transgenic line-T4 flowered with 50 days after transplanting, while wild-type (WT) plants flowered with 65 days after transplanting. Over-expression of *PtNF-YB1* promoted early flowering in transgenic tomato, indicating that its ability to promote early flowering can cross species barriers. Therefore, the poplar *PtNF-YB1* may serve as a potential candidate gene for improve early flowering of poplar and other crops through genetic transformation.

### Regulation of flowering pathway genes in the transgenic *Arabidopsis* and the potential molecular mechanism model for how *PtNF-YB1* expression can promote early flowering in poplar

**Fig. 5:**
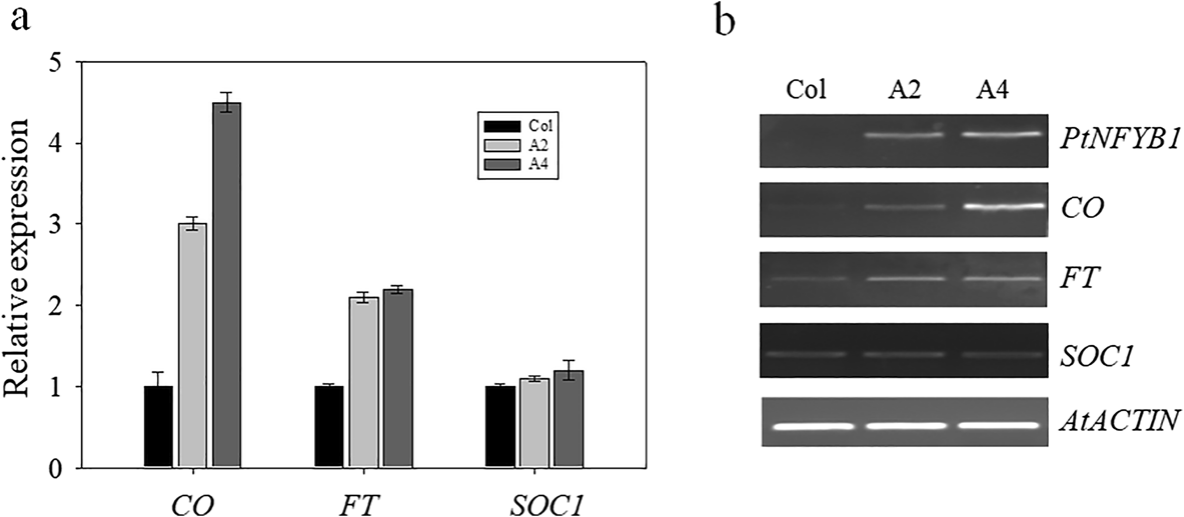
The expression analysis of known flowering genes in transgenic *Arabidopsis.* **a** Expression analysis of flowering genes with qRT–PCR. **b** Expression analysis of flowering geneswith semi-quantitative RT-RCRs. The three known flowering genes were *CO*, *FT* and *SOC1*. The RT-PCR reactions were repeated three times

How does *PtNF-YB1* regulate the mechanism of early flowering in poplar? To understand this mechanisms, the expression levels of *Arabidopsis* three known flowering genes (*CO*, *FT* and *SOC1*) were examined with RT-PCR in the wild-type (Col) and transgenic plants (A2 and A4) (Fig. 5). Among of them, two genes were up-regulated, including *Arabidopsis CONSTANS* (*CO*) and *FT*. The CO is a key regulator of photoperiod-dependent flowering time in *Arabidopsis* [43]*.* The FT acts partially downstream of CO, which promotes flowering in plants [44, 45]. The *SOC1* gene showed no difference between wild-type (Col) and transgenic plants. The *SOC1* gene is a MADS transcription factor, a key integrator in photoperiod pathway [46]. This result was consistent with previous findings [38].

**Fig. 6:**
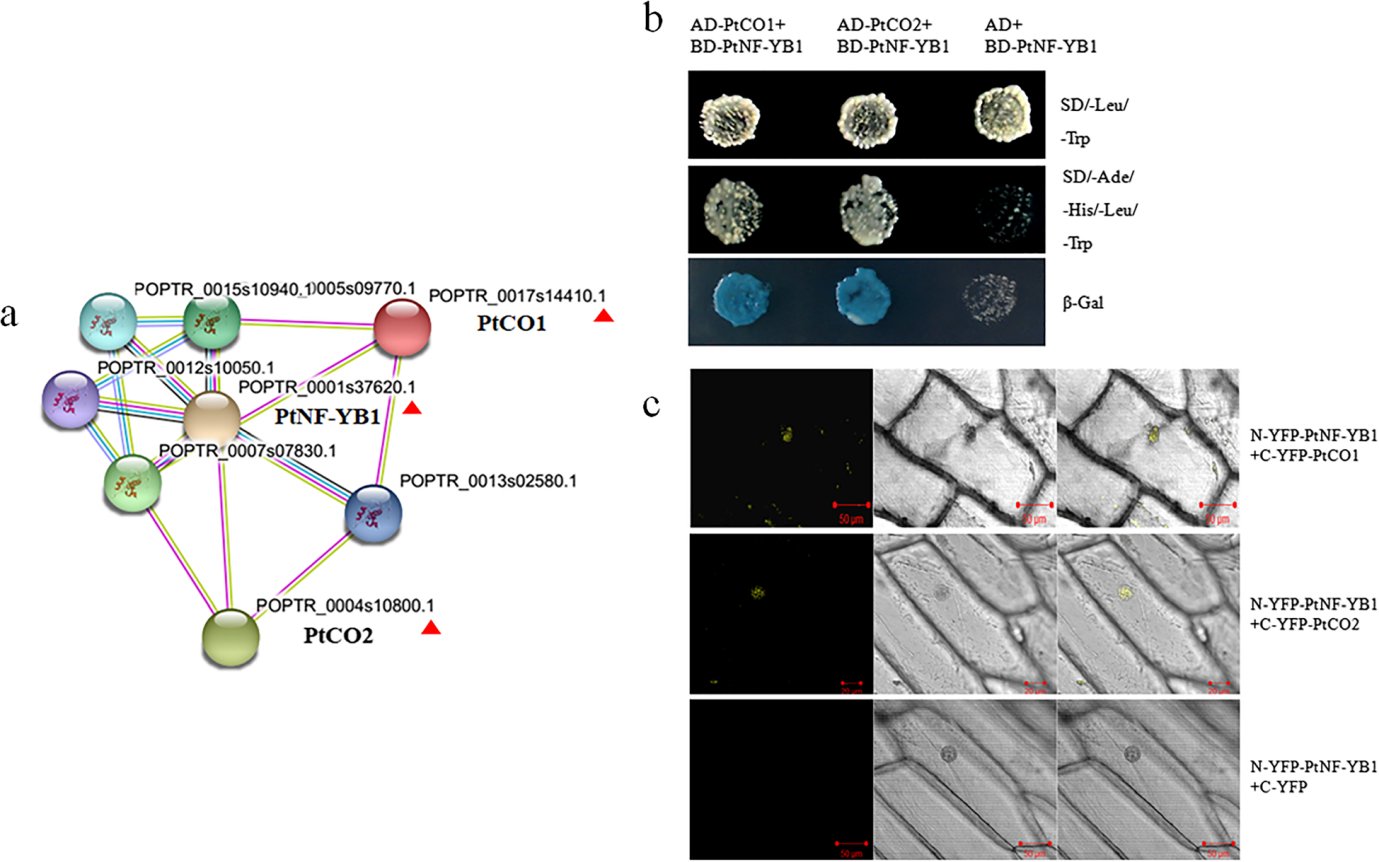
PtNF-YB1 protein interactions with PtCO1 and PtCO2. **a** The potential PtNF-YB1 protein interactions with PtCO1 and PtCO2 were predicted using STRING software. **b** Y2H assays showing protein–protein interactions. **c** BiFC assay to detect the interactions of proteins

**Fig. 7:**
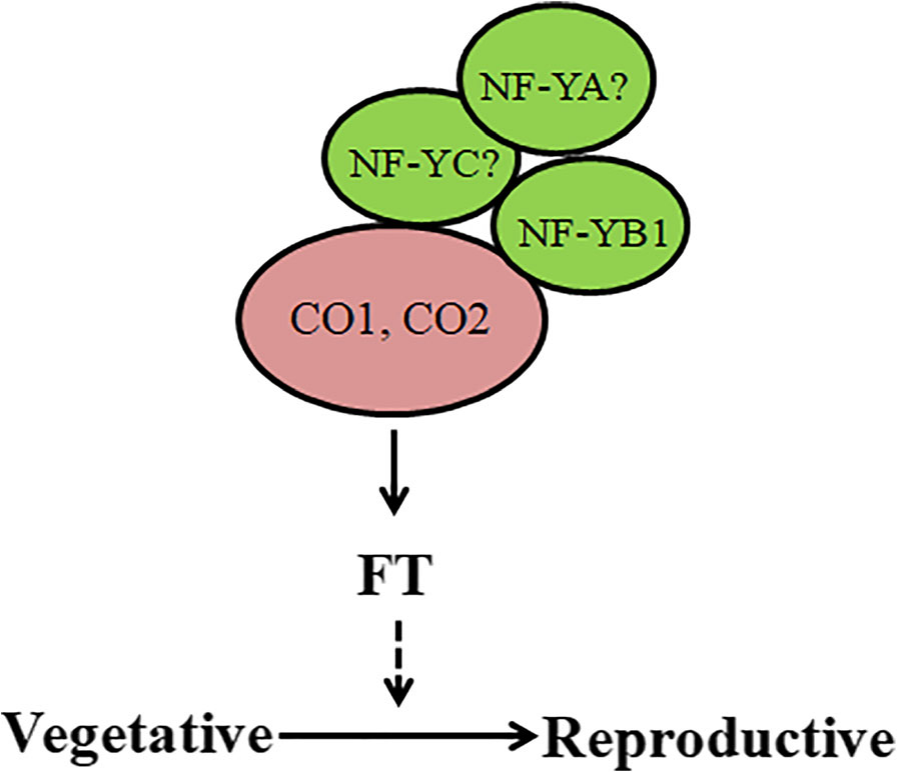
A potential molecular mechanism model for how PtNF-YB1 expression can promote early flowering in poplar

For the study of poplar early flowering, the main focus is on the study *Arabidopsis* homologue gene CO/FT [1, 2, 47–49]. Through the study of the model plant *Arabidopsis*, it was shown that AtNF-YB2 and AtCO interact to regulate FT and promote early flowering [27, 38]. In poplar, two CO-like genes PtCO1 (POPTR0017s14410.1) and PtCO2 (POPTR0004s10800.1) are the closest structural orthologs of AtCO (At5g15840) (Additional file 3). The protein string interactions suggest a possible link between PtNF-YB1 and PtCO (PtCO1 and PtCO2) in poplar (Fig. 6a). We used the Y2H and BiFC assays to validate these hypotheses interactions between PtNF-YB1 and PtCO (PtCO1 and PtCO2) proteins in poplar (Fig. 6b, c). The poplar PtNF-YB1 promotion of flowering is achieved probably by interacting with PtCO1 and PtCO2 proteins (Fig. 7). We also generated *PtNF-YB1* over-expressing transgenic poplar. So far, the transgenic poplar did not show the expected early flowering (Additional file 4). There may be at least three reasons to explain this phenomenon: (i) Epigenetic mechanism. Previous evidence supported that the NF-Y transcription factor as important modulators of epigenetic marks controlling flowering [50–53]. (ii) The multiple-year delay in onset of flowering of woody perennials. (iii) Whether PtNF-YA/PtNF-YC are involved in the formation of PtNF-Y complexes to regulate poplar flowering. Our future work is needed to analyze these questions through epigenetics and proteomics.

In summary, to elucidate the role of NF-Y transcription factor in poplar flowering induction and molecular regulation mechanism will be important for people to understand the role and function of NF-Y transcription factor family in woody plants, and provide important theoretical basis for regulating flowering time and shortening breeding cycle.

## Conclusions

In the present study, we have identified the poplar *NF-YB* gene family and confirmed the function of the *PtNF-YB1* regulate flowering timing using transgenic *Arabidopsis* and tomato***.*** To understand this mechanisms, three known flowering genes (*CO*, *FT* and *SOC1*) were examined by RT-PCR in transgenic *Arabidopsis*. We also used the Y2H and BiFC to assay the interactions between poplar PtNF-YB1 and PtCO (PtCO1 and PtCO2) proteins. A potential molecular mechanism model in which *PtNF-YB1* play a role in regulating flowering in poplar was discussed. Therefore, *PtNF-YB1* can be used as a potential candidate gene to improve early flowering by genetic transformation in poplar and other crops.

## Methods

### Identification PtNF-YB family members in poplar

The *Arabidopsis* and rice NF-YB sequences were retrieved from the *Arabidopsis* TAIR database (https://www.arabidopsis.org) and rice OrygenesDB database (http://orygenesdb.cirad.fr/), respectively. The BLASTN program was used with an E-value cut-off of 1.0e^− 5^ to identify predicted PtNF-YB sequences using Phytozome database *Populus trichocarpa* V3.0 (https://phytozome.jgi.doe.gov/pz/portal.html).

### Phylogenetic trees and conserved motif analyses

The phylogenetic trees were constructed by the MEGA V5.5 Neighbor-Joining (NJ) method using conserved and amino acid sequences, and the parameters were p-distance model and 1000 bootstrap replicates. Multiple sequence alignments were implemented by Clustal X software. The conserved motifs of 21 poplar PtNF-YBs were analyzed using the Multiple Expectation Maximization for Motif Elicitation (MEME V4.12.0) (http://meme-suite.org/tools/meme) by uploading the coding sequences according the instructions.

### Plant material and growth conditions

The 6-year-old poplar 84K flowering (F), foral buds (FB), root (R), stem (S) and leaf (L) were collected from the Wei River planting base in Xi’an city (N33°42′44.37″; E 107°39′36.62″; with altitude 500–550 m), Shannxi province, China. For transformation, wild-type *Arabidopsis* ecotype columbia (Col) were used. It was grown in the long day conditions (LD, 16 h light/8 h dark) at 20–22 °C. For tomato genetic transformation, “Micro-Tom” tomato were used as the method described by Zhang and Blumwald [54]. It was grown in the nursery soils pots and the greenhouse conditions at day (25 °C) and night (20 °C).

### *PtNF-YB1* over-expressing vector construction

The open reading frame (ORF) of *PtNF-YB1* gene were amplified by RT-PCR, and then was used to construct over-expression vector. The *PtNF-YB1* gene was inserted into the vector pBI121 and under the 35 S promoter of the cauliflower mosaic virus (CaMV). The specific primers were shown in Additional file 5.

### *Arabidopsis* and tomato transformation

The poplar *PtNF-YB1* over-expressing constructs was introduced into Col with a floral dip method mediated with *Agrobacterium* strain GV3101 [55]. The seeds of positive transgenic plants carrying the *PtNF-YB1* constructs were individually harvested. Homozygous transgenic lines were used for further investigation. “Micro-Tom” tomato cotyledons were transformed with the *Agrobacterium* strain LBA4404 containing the *PtNF-YB1* over-expressing constructs as the method described by Zhang and Blumwald [54].

### Yeast two-hybrid (Y2H) assay

According to the manufacturer’s instructions (Clontech, USA), we performed yeast two-hybrid (Y2H) experiments using a Gal4-based two-hybrid system. First, the poplar *PtNF-YB1* gene ORF was inserted into the bait vector pGBKT7. The resulting vector pGBKT7-PtNF-YB1 was used as a bait. The ORFs of *PtNF-CO1* and *PtNF-CO2* genes were cloned into the vector pGADT7. The specific primers are shown in Additional file 6. Then, co-transformation of pGADT7 with pGBKT7-PtNF-YB1 was used as a control, the pGBKT7-PtNF-YB1 construct was used together with pGADT7-PtNF-CO1 and pGADT7-PtNF-CO2 to co-transform the yeast strain AH109. Finally, positive colonies were selected using SD/−Trp-Leu-His-Ade medium and stained with *β*- galactosidase to confirm the positive colonies.

### Bimolecular fluorescence complementation (BiFC) assay

We used the vectors pSPYNE-35S and pSPYCE-35S and the cotransfection vector 35S: P19 to construct a bimolecular fluorescent complementary (BiFC) plasmid vector. For the first time, the poplar *PtNF-YB1* gene ORF was inserted into the vector pSPYNE-35S and the *PtCO* (*PtCO1* and *PtCO2*) gene ORF were inserted into the vector pSPYCE-35S. Both the vectors contain the N- or C-terminus encoding the yellow fluorescent protein (YFP). The specific primers are shown in Additional file 7. Then, as described by Walter et al., we used the *Agrobacterium*-mediated infection method to introduce different combinations of gene vectors into onion epidermal cells [56]. Finally, the expression of YFP in onion epidermal cells was observed using a laser confocal microscope (Zeiss LSM510 Meta, Germany) after 48 h incubation at 24 °C. We use a wavelength of 488 nm and detection at 500–530 nm with a band-path filter for YFP.

### Reverse transcription PCR (RT-PCR)

Semi-quantitative reverse transcription PCR (RT-PCR) was used to detect the expression level of *PtNF-YB1* in poplar, *Arabidopsis* and tomato. Quantitative real-time reverse transcription PCR (RT-qPCR) were performed to confirm the results. The RT-qPCR reactions were performed in a Step One Plus Real-Time PCR System (Applied Biosystems, USA) using a Super Real PreMix kit (SYBR Green) (Tiangen-biotech, China). The RNA relative expression of each gene was calculated according to the 2^-ΔΔCT^ method, as reported previously in detail [57]. In RT-qPCR analysis, the *18S rRNA* (poplar), *AtACTIN* (*Arabidopsis*) and *T-Act* (tomato) as the internal control gene. The RT-PCR reactions were repeated three times. The specific primers were shown in Additional files 8 and 9.

## Additional files

Additional file 1: The phylogenetic tree and conserved motifs analysis of NF-YB families in poplar. a. PtNF-YBs phylogenetic tree. b. PtNF-YBs conserved motifs analysis. (TIF 2879 kb)

Additional file 2: Flowering time of *Arabidopsis* transgenic lines ectopically expressing *PtNF-YB1*. (DOC 29 kb)

Additional file 3: Analysis of the deduced amino acid sequence of poplar and *Arabidopsis* CO. a. The homology tree of poplar PtCO1, PtCO2 and AtCO. b. Multiple sequences alignment of the conversed domains PtCO1, PtCO2 and AtCO. The amino acid sequences were analyzed using DNAMAN software. (TIF 786 kb)

Additional file 4: **Figure S3.** Over-expressing *PtNF-YB1* in transgenic poplar lines. a. The wild-type (WT) and transgenic tomato lines (PT1, PT3 and PT4) 45 days after transfer to the growth chamber. Bar = 10 cm. b. The wild-type (WT1 and WT2) and transgenic tomato lines ((PT1 and PT3) 80 days after transfer to the growth chamber. Bar = 22 cm. (TIF 1503 kb)

Additional file 5: Primers for *PtNF-YB1* gene cloning and over-expressing vector construction. (DOC 28 kb)

Additional file 6: Primers for yeast two-hybrid (Y2H) assay. (DOC 28 kb)

Additional file 7: Primers for bimolecular fluorescence complementation (BiFC) assay. (DOC 28 kb)

Additional file 8: Primers for expression analysis using semi-quantitative RT-PCR. (DOC 32 kb)

Additional file 9: Primers for expression analysis using qRT-PCR. (DOC 33 kb)

## Data Availability

All data and materials supporting the results of this study are included in the article and the additional files.

## Abbreviations

BiFC: Bimolecular fluorescence complementation
CaMV: Cauliflower mosaic virus
HFM: Histone-fold motif
MW: Molecular weight
NF-Y: Nuclear factor Y
NJ: Neighbor-Joining
ORF: Open reading frame
PI: Isoelectric point
RT-PCR: Semi-quantitative reverse transcription PCR
RT-qPCR: Quantitative real-time reverse transcription PCR
Y2H: Yeast two-hybrid

## References

[1] Hsu CY, Adams JP, Kim H, No K, Ma C, Strauss SH, Drnevich J, Vandervelde L, Ellis JD, Rice BM (2011). FLOWERING LOCUS T duplication coordinates reproductive and vegetative growth in perennial poplar. Proc Natl Acad Sci.

[2] Hsu CY, Liu Y, Luthe DS, Yuceer C (2006). Poplar FT2 shortens the juvenile phase and promotes seasonal flowering. Plant Cell.

[3] Petersen R, Krost C (2013). Tracing a key player in the regulation of plant architecture: the columnar growth habit of apple trees (*Malus x domestica*). Planta.

[4] Petterle A, Karlberg A, Bhalerao RP (2013). Daylength mediated control of seasonal growth patterns in perennial trees. Curr Opin Plant Biol.

[5] Flachowsky H, Hanke MV, Peil A, Strauss SH, Fladung M (2009). A review on transgenic approaches to accelerate breeding of woody plants. Plant Breed.

[6] Edwards D, Murray JA, Smith AG (1998). Multiple genes encoding the conserved CCAAT-box transcription factor complex are expressed in *Arabidopsis*. Plant Physiol.

[7] Siefers N, Dang KK, Kumimoto RW, WEt B, Tayrose G, Holt BF (2009). Tissue-specific expression patterns of Arabidopsis NF-Y transcription factors suggest potential for extensive combinatorial complexity. Plant Physiol.

[8] Maruyama K, Todaka D, Mizoi J, Yoshida T, Kidokoro S, Matsukura S, Takasaki H, Sakurai T, Yamamoto YY, Yoshiwara K (2012). Identification of cis-acting promoter elements in cold- and dehydration-induced transcriptional pathways in *Arabidopsis*, rice, and soybean. DNA Res.

[9] Dolfini D, Gatta R, Mantovani R (2012). NF-Y and the transcriptional activation of CCAAT promoters. Crit Rev Biochem Mol Biol.

[10] Thirumurugan T, Ito Y, Kubo T, Serizawa A, Kurata N (2008). Identification, characterization and interaction of HAP family genes in rice. Mol Gen Genomics.

[11] Mei X, Liu C, Yu T, Liu X, Xu D, Wang J, Wang G, Cai Y (2015). Identification and characterization of paternal-preferentially expressed gene *NF-YC8* in maize endosperm. Mol Gen Genomics.

[12] Zhai L, Xu L, Wang Y, Zhu X, Feng H, Li C, Luo X, Everlyne MM, Liu L (2016). Transcriptional identification and characterization of differentially expressed genes associated with embryogenesis in radish (*Raphanus sativus* L). Sci Rep.

[13] Yazawa K, Kamada H (2007). Identification and characterization of carrot HAP factors that form a complex with the embryo-specific transcription factor C-LEC1. J Exp Bot.

[14] Mu J, Tan H, Zheng Q, Fu F, Liang Y, Zhang J, Yang X, Wang T, Chong K, Wang XJ (2008). LEAFY COTYLEDON1 is a key regulator of fatty acid biosynthesis in *Arabidopsis*. Plant Physiol.

[15] Junker A, Monke G, Rutten T, Keilwagen J, Seifert M, Thi TM, Renou JP, Balzergue S, Viehover P, Hahnel U (2012). Elongation-related functions of LEAFY COTYLEDON1 during the development of *Arabidopsis thaliana*. Plant J.

[16] Stephenson TJ, McIntyre CL, Collet C, Xue GP (2007). Genome-wide identification and expression analysis of the NF-Y family of transcription factors in *Triticum aestivum*. Plant Mol Biol.

[17] Stephenson TJ, Mcintyre CL, Collet C, Xue GP (2010). *TaNF-YC11*, one of the light-upregulated NF-YC members in *Triticum aestivum*, is co-regulated with photosynthesis-related genes. Funct Integr Genomics.

[18] Stephenson TJ, Mcintyre CL, Collet C, Xue GP (2011). *TaNF-YB3* is involved in the regulation of photosynthesis genes in *Triticum aestivum*. Functional & Integrative Genomics.

[19] Combier JP, Frugier F, De BF, Boualem A, El-Yahyaoui F, Moreau S, Vernié T, Ott T, Gamas P, Crespi M (2006). *MtHAP2-1* is a key transcriptional regulator of symbiotic nodule development regulated by microRNA169 in *Medicago truncatula*. Genes Dev.

[20] Nelson DE, Repetti PP, Adams TR, Creelman RA, Wu J, Warner DC, Anstrom DC, Bensen RJ, Castiglioni PP, Donnarummo MG (2007). Plant nuclear factor Y (NF-Y) B subunits confer drought tolerance and lead to improved corn yields on water-limited acres. Proc Natl Acad Sci U S A.

[21] Li WX, Oono YJ, He XJ, Wu JM, Iida K, Lu XY, Cui X, Jin H, Zhu JK (2008). The *Arabidopsis NFYA5* transcription factor is regulated transcriptionally and Posttranscriptionally to promote drought resistance. Plant Cell.

[22] Liu JX, Howell SH (2010). bZIP28 and NF-Y transcription factors are activated by ER stress and assemble into a transcriptional complex to regulate stress response genes in *Arabidopsis*. Plant Cell.

[23] Yan DH, Xia X, Yin W (2013). NF-YB family genes identified in a poplar genome-wide analysis and expressed in *Populus euphratica* are responsive to drought stress. Plant Mol Biol Report.

[24] Palmeros-Suárez PA, Massange-Sánchez JA, Martínez-Gallardo NA, Montero-Vargas JM, Gómez-Leyva JF, Délano-Frier JP (2015). The overexpression of an *Amaranthus hypochondriacus* NF-YC gene modifies growth and confers water deficit stress resistance in *Arabidopsis*. Plant Science An International Journal of Experimental Plant Biology.

[25] Xuanyuan G, Lu C, Zhang R, Jiang J (2017). Overexpression of *StNF-YB3.1* reduces photosynthetic capacity and tuber production, and promotes ABA-mediated stomatal closure in potato (*Solanum tuberosum L.*). Plant Sci.

[26] Ben-Naim Orna, Eshed Ravit, Parnis Anna, Teper-Bamnolker Paula, Shalit Akiva, Coupland George, Samach Alon, Lifschitz Eliezer (2006). The CCAAT binding factor can mediate interactions between CONSTANS-like proteins and DNA. The Plant Journal.

[27] Cai X, Ballif J, Endo S, Davis E, Liang M, Chen D, Dewald D, Kreps J, Zhu T, Wu Y (2007). A putative CCAAT-binding transcription factor is a regulator of flowering timing in *Arabidopsis*. Plant Physiol.

[28] Chen NZ, Zhang XQ, Wei PC, Chen QJ, Ren F, Chen J, Wang XC (2007). *AtHAP3b* plays a crucial role in the regulation of flowering time in *Arabidopsis* during osmotic stress. J Biochem Mol Biol.

[29] Cao S, Kumimoto RW, Gnesutta N (2014). A distal CCAAT/NUCLEAR FACTOR Y complex promotes chromatin looping at the FLOWERING LOCUS T promoter and regulates the timing of flowering in *Arabidopsis*. Plant Cell.

[30] Hackenberg D, Wu Y, Voigt A, Adams R, Schramm P, Grimm B (2012). Studies on differential nuclear translocation mechanism and assembly of the three subunits of the *Arabidopsis thaliana* transcription factor NF-Y. Mol Plant.

[31] Liang M, Hole D, Wu J, Blake T, Wu Y (2012). Expression and functional analysis of *NUCLEAR FACTOR-Y*, subunit B genes in barley. Planta.

[32] Liang M, Yin X, Lin Z, Zheng Q, Liu G, Zhao G (2014). Identification and characterization of NF-Y transcription factor families in canola (*Brassica napus* L.). Planta.

[33] Kumimoto RW, Zhang Y, Siefers N, Holt BF (2010). NF-YC3, NF-YC4 and NF-YC9 are required for CONSTANS-mediated, photoperiod-dependent flowering in *Arabidopsis thaliana*. Plant J.

[34] Siriwardana CL, Gnesutta N, Kumimoto RW, Jones DS, Myers ZA, Mantovani R, Rd HBNUCLEARFACTORY, Subunit A (2016). (NF-YA) proteins positively regulate flowering and act through *FLOWERING LOCUS T*. PLoS Genet.

[35] Wei X, Xu J, Guo H, Jiang L, Chen S, Yu C, Zhou Z, Hu P, Zhai H, Wan J (2010). DTH8 suppresses flowering in rice, influencing plant height and yield potential simultaneously. Plant Physiol.

[36] Yan WH, Wang P, Chen HX, Zhou HJ, Li QP, Wang CR, Ding ZH, Zhang YS, Yu SB, Xing YZ (2011). A major QTL, *Ghd8*, plays pleiotropic roles in regulating grain productivity, plant height, and heading date in rice. Mol Plant.

[37] Dai X, Ding Y, Tan L, Fu Y, Liu F, Zhu Z, Sun X, Sun X, Gu P, Cai H (2012). *LHD1*, an allele of *DTH8/Ghd8*, controls late heading date in common wild rice (*Oryza rufipogon*). J Integr Plant Biol.

[38] Kumimoto RW, Adam L, Hymus GJ, Repetti PP, Reuber TL, Marion CM, Hempel FD, Ratcliffe OJ (2008). The nuclear factor Y subunits NF-YB2 and NF-YB3 play additive roles in the promotion of flowering by inductive long-day photoperiods in *Arabidopsis*. Planta.

[39] Maity SN, De CB (1992). Biochemical analysis of the B subunit of the heteromeric CCAAT-binding factor. A DNA-binding domain and a subunit interaction domain are specified by two separate segments. J Biol Chem.

[40] Sinha S, Kim IS, Sohn KY, de Crombrugghe B, Maity SN (1996). Three classes of mutations in the a subunit of the CCAAT-binding factor CBF delineate functional domains involved in the three-step assembly of the CBF-DNA complex. Mol Cell Biol.

[41] Romier C, Cocchiarella F, Mantovani R, Moras D (2003). The NF-YB/NF-YC structure gives insight into DNA binding and transcription regulation by CCAAT factor NF-Y. J Biol Chem.

[42] Mantovani R (1999). The molecular biology of the CCAAT-binding factor NF-Y. Gene.

[43] Putterill J, Robson F, Lee K, Simon R, Coupland G (1995). The CONSTANS gene of *Arabidopsis* promotes flowering and encodes a protein showing similarities to zinc finger transcription factors. Cell.

[44] Kardailsky I, Shukla VK, Ahn JH, Dagenais N, Christensen SK, Nguyen JT, Chory J, Harrison MJ, Weigel D (1999). Activation tagging of the floral inducer FT. Science.

[45] Kobayashi Y, Kaya H, Goto K, Iwabuchi M, Araki T (1999). A pair of related genes with antagonistic roles in mediating flowering signals. Science.

[46] Moon J, Suh SS, Lee H, Choi KR, Hong CB, Paek NC, Kim SG, Lee I (2003). The SOC1 MADS-box gene integrates vernalization and gibberellin signals for flowering in *Arabidopsis*. Plant J.

[47] Bohlenius H, Huang T, Charbonnel-Campaa L, Brunner AM, Jansson S, Strauss SH, Nilsson O (2006). *CO/FT* regulatory module controls timing of flowering and seasonal growth cessation in trees. Science.

[48] Rinne PLH, Schoot CVD (2011). Chilling of dormant buds hyperinduces *FLOWERING LOCUS T* and recruits GA-inducible 1,3-β-Glucanases to reopen signal conduits and release dormancy in *Populus*. Plant Cell.

[49] Shen L, Chen Y, Su X, Zhang S, Pan H, Huang M (2012). Two FT orthologs from *Populus simonii* Carrière induce early flowering in *Arabidopsis* and poplar trees. Plant Cell Tissue & Organ Culture.

[50] Zicola J, Liu L, Tanzler P, Turck F (2019). Targeted DNA methylation represses two enhancers of *FLOWERING LOCUS T* in *Arabidopsis thaliana*. Nature plants.

[51] Zhang H, Zhu S, Liu T, Wang C, Cheng Z, Zhang X, Chen L, Sheng P, Cai M, Li C (2019). DELAYED HEADING DATE1 interacts with OsHAP5C/D, delays flowering time and enhances yield in rice. Plant Biotechnol J.

[52] Myers ZA, Holt BF (2018). NUCLEAR FACTOR-Y: still complex after all these years? Current opinion in plant biology.

[53] Liu X, Yang Y, Hu Y, Zhou L, Li Y, Hou X (2018). Temporal-specific interaction of NF-YC and CURLY LEAF during the floral transition regulates flowering. Plant Physiol.

[54] Zhang HX, Blumwald E (2001). Transgenic salt-tolerant tomato plants accumulate salt in foliage but not in fruit. Nat Biotechnol.

[55] Clough SJ, Bent AF (1998). Floral dip: a simplified method for *Agrobacterium*-mediated transformation of *Arabidopsis thaliana*. Plant J.

[56] Walter M, Chaban C, Schutze K, Batistic O, Weckermann K, Nake C, Blazevic D, Grefen C, Schumacher K, Oecking C, Harter K, Kudla J (2004). Visualization of protein interactions in living plant cells using bimolecular fluorescence complementation. Plant J.

[57] Livak KJ, Schmittgen TD (2001). Analysis of relative gene expression data using real-time quantitative PCR and the 2^-ΔΔCT^ method. Methods.

