# Supplementary figures and images for: Genome-wide analysis of poplar NF-YB gene family and identified PtNF-YB1 important in regulate flowering timing in transgenic plants
Source: BMC Plant Biol. 2019 Jun 11;19:251. doi: 10.1186/s12870-019-1863-2 (PMC6560884; doi:10.1186/s12870-019-1863-2)

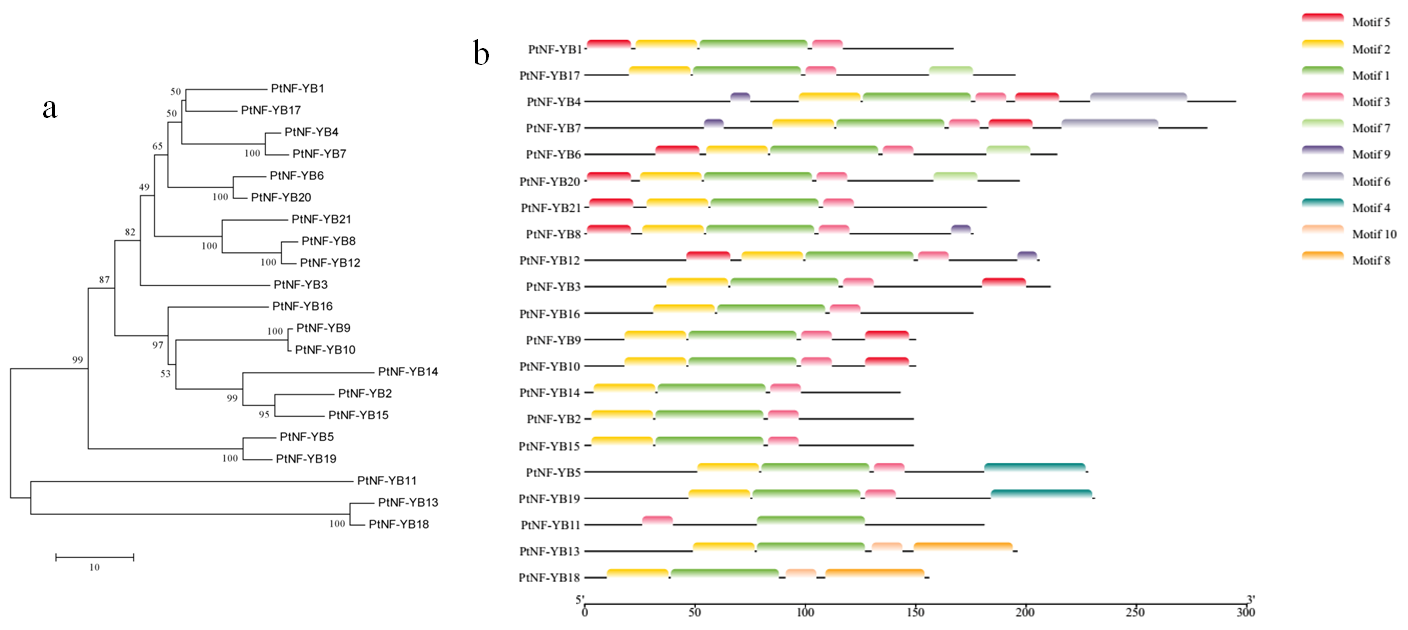

Supplement: Supplementary file 1 — The phylogenetic tree and conserved motifs analysis of NF-YB families in poplar. a. PtNF-YBs phylogenetic tree. b. PtNF-YBs conserved motifs analysis. (TIF 2879 kb) [file 12870_2019_1863_MOESM1_ESM.tif]

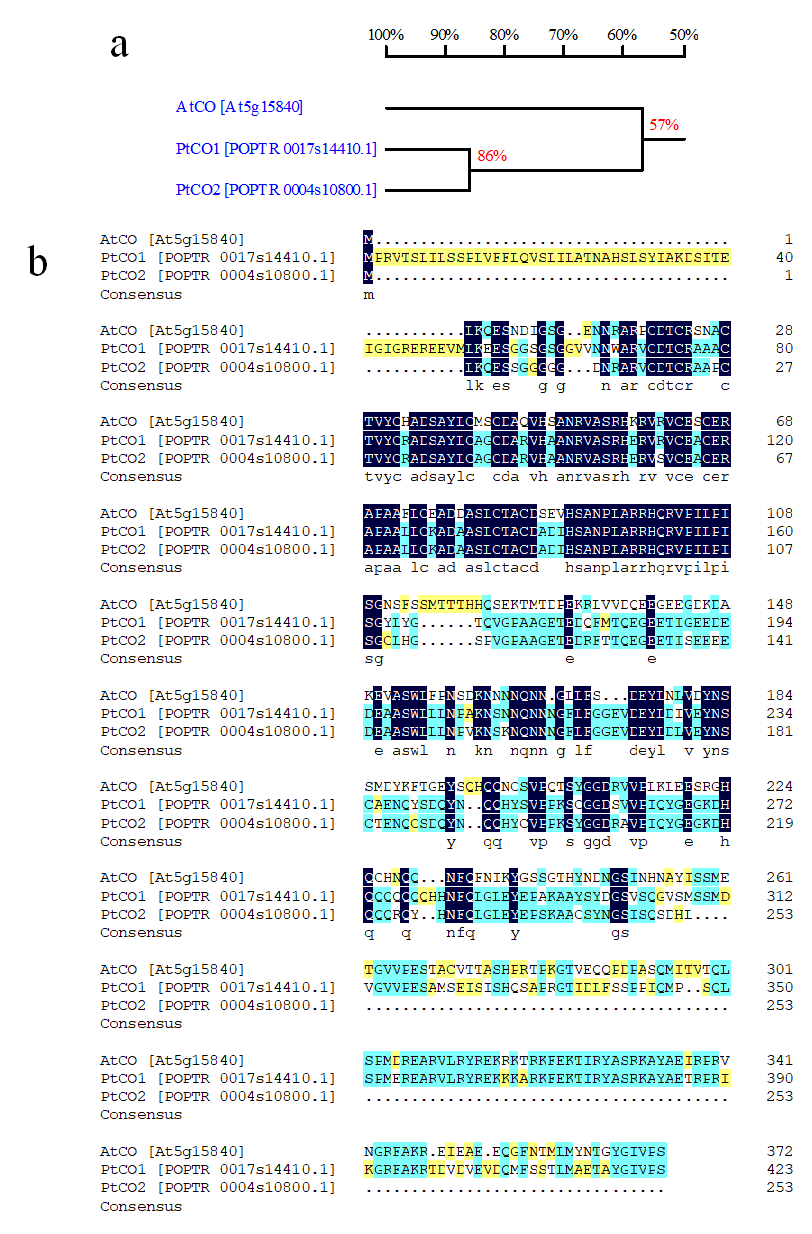

Supplement: Supplementary file 3 — Analysis of the deduced amino acid sequence of poplar and Arabidopsis CO. a. The homology tree of poplar PtCO1, PtCO2 and AtCO. b. Multiple sequences alignment of the conversed domains PtCO1, PtCO2 and AtCO. The amino acid sequences were analyzed using DNAMAN software. (TIF 786 kb) [file 12870_2019_1863_MOESM3_ESM.tif]

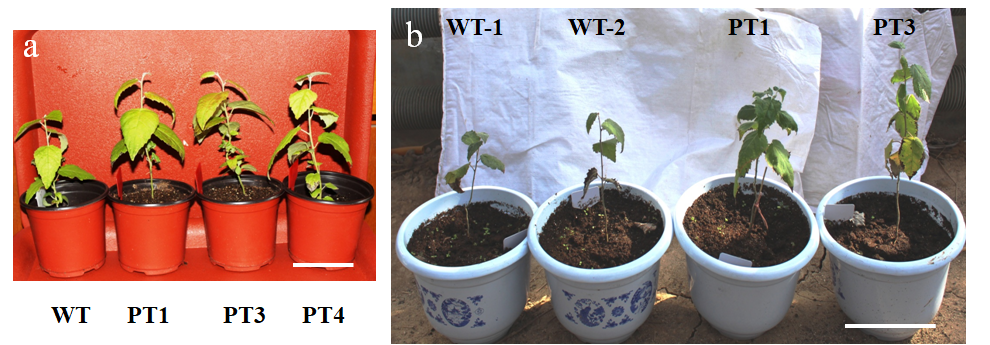

Supplement: Supplementary file 4 — Figure S3. Over-expressing PtNF-YB1 in transgenic poplar lines. a. The wild-type (WT) and transgenic tomato lines (PT1, PT3 and PT4) 45 days after transfer to the growth chamber. Bar = 10 cm. b. The wild-type (WT1 and WT2) and transgenic tomato lines ((PT1 and PT3) 80 days after transfer to the growth chamber. Bar = 22 cm. (TIF 1503 kb) [file 12870_2019_1863_MOESM4_ESM.tif]
