# Supplementary material for: Genome-wide analysis of poplar NF-YB gene family and identified PtNF-YB1 important in regulate flowering timing in transgenic plants
Source: BMC Plant Biol. 2019 Jun 11;19:251. doi: 10.1186/s12870-019-1863-2 (PMC6560884; doi:10.1186/s12870-019-1863-2)
Supplement: Supplementary file 2 — Flowering time of Arabidopsis transgenic lines ectopically expressing PtNF-YB1. (DOC 29 kb) [file 12870_2019_1863_MOESM2_ESM.doc]

**Additional file 2: Flowering time of *Arabidopsis* transgenic lines ectopically expressing *PtNF-YB1***

| Line | LD conditions (16 h light/8 h dark) | | |
| --- | --- | --- | --- |
|  | No. of plants | Rosette leaves | Cauline leaves |
| Col | 25 | 12.5±0.4 | 5.1±0.5 |
| A2 | 15 | 7.3±0.2 | 3.6±0.5 |
| A4 | 16 | 7.1±0.4 | 3.4±0.6 |
