# Supplementary material for: Genome-wide analysis of poplar NF-YB gene family and identified PtNF-YB1 important in regulate flowering timing in transgenic plants
Source: BMC Plant Biol. 2019 Jun 11;19:251. doi: 10.1186/s12870-019-1863-2 (PMC6560884; doi:10.1186/s12870-019-1863-2)
Supplement: Supplementary file 5 — Primers for PtNF-YB1 gene cloning and over-expressing vector construction. (DOC 28 kb) [file 12870_2019_1863_MOESM5_ESM.doc]

**Additional file 5:** Primers for *PtNF-YB1* gene cloning and over-expressing vector construction

| Constructions | Primers |
| --- | --- |
| PtNF-YB1 | forward: 5’-ATGGCGGACTCAGACAACGAC-3’  reverse: 5’-CTATCTGAGCCTACCCAAGCTATCTCC-3’ |
