# Supplementary material for: Genome-wide analysis of poplar NF-YB gene family and identified PtNF-YB1 important in regulate flowering timing in transgenic plants
Source: BMC Plant Biol. 2019 Jun 11;19:251. doi: 10.1186/s12870-019-1863-2 (PMC6560884; doi:10.1186/s12870-019-1863-2)
Supplement: Supplementary file 6 — Primers for yeast two-hybrid (Y2H) assay. (DOC 28 kb) [file 12870_2019_1863_MOESM6_ESM.doc]

**Additional file 6** Primers for yeast two-hybrid (Y2H) assay

| Constructions | Primers |
| --- | --- |
| pGBKT7-PtNF-YB1 | forward: 5’-ATGGCGGACTCAGACAACGAC-3’  reverse: 5’-TCTGAGCCTACCCAAGCTATCTCC-3’ |
| pGADT7-PtNF-CO1 | forward: 5’-ATGCCACGTGTCACATCCTTGAT-3’  reverse: 5’-GAATGATGGGACAATGCCATATGCT-3’ |
| pGADT7-PtNF-CO2 | forward: 5’-ATGTTGAAGCAAGAGAGTAGTGGTGG-3’  reverse: 5’-GAGATGATCACTTTGACTGATCGAACCA-3’ |
