# Supplementary material for: Genome-wide analysis of poplar NF-YB gene family and identified PtNF-YB1 important in regulate flowering timing in transgenic plants
Source: BMC Plant Biol. 2019 Jun 11;19:251. doi: 10.1186/s12870-019-1863-2 (PMC6560884; doi:10.1186/s12870-019-1863-2)
Supplement: Supplementary file 8 — Primers for expression analysis using semi-quantitative RT-PCR. (DOC 32 kb) [file 12870_2019_1863_MOESM8_ESM.doc]

**Additional file 8:** Primers for expression analysis using semi-quantitative RT-PCR

| Gene name | Primers |
| --- | --- |
| *PtNF-YB1* | forward: 5’-ATGGCGGACTCAGACAACGAC-3’  reverse: 5’-CCTGTCACGCGCCACGGTATTCT-3’ |
| *18S rRNA* | forward: 5’-GGAATTGACGGAAGGGCACCACCAGGC-3’  reverse: 5’-GGACATCTAAGGGCATCACAGACCTG -3’ |
| *AtACTIN* | forward: 5’-GGAAAGGATCTGTACGGTAAC-3’  reverse: 5’-TGTGAACGATTCCTGGAC-3’ |
| *T-Act* | forward: 5’-CTTCAGTCCACAATCGGTGG-3’  reverse: 5’-CATTCCGAGTTGAGCTGCTG-3’ |
| *CO* | forward: 5’-GACTGGTGGTGGATCAAGAGGAAGGTGAAG -3’  reverse: 5’-CATCGTGTTGAACCCTTGCTCCTCGGCTTC-3’ |
| *FT* | forward: 5’-TTGTTGGAGACGTTCTTGATCCGTTTAATAG-3’  reverse: 5’-CCTCCGCAGCCACTCTCCCTCTGACAATTG -3’ |
| *SOC1* | forward: 5’-GGCATACTAAGGATCGAGTCAGCACCAAAC-3’  reverse: 5’-ACCCAATGAACAATTGCGTCTCTACTTCAG -3’ |
