# Supplementary material for: Genome-wide analysis of poplar NF-YB gene family and identified PtNF-YB1 important in regulate flowering timing in transgenic plants
Source: BMC Plant Biol. 2019 Jun 11;19:251. doi: 10.1186/s12870-019-1863-2 (PMC6560884; doi:10.1186/s12870-019-1863-2)
Supplement: Supplementary file 9 — Primers for expression analysis using qRT-PCR. (DOC 33 kb) [file 12870_2019_1863_MOESM9_ESM.doc]

**Additional file 9:** Primers for expression analysis using qRT-PCR

| Gene name | Primers |
| --- | --- |
| *PtNF-YB1* | forward: 5’-ATGGCGGACTCAGACAACGAC-3’  reverse: 5’-GCTCCACGTATTCCTCAAAGCC-3’ |
| *18S rRNA* | forward: 5’- GGAATTGACGGAAGGGCACCACCAGGC-3’  reverse: 5’- GGACATCTAAGGGCATCACAGACCTG -3’ |
| *AtACTIN* | forward: 5’-GGAAAGGATCTGTACGGTAAC-3’  reverse: 5’-TGTGAACGATTCCTGGAC-3’ |
| *T-Act* | forward: 5’-CTTCAGTCCACAATCGGTGG-3’  reverse: 5’-CATTCCGAGTTGAGCTGCTG-3’ |
| *CO* | forward: 5’-CAGGCAAACAGTGTATGCACCAGG -3’  reverse: 5’-CCGCAGCCACTCTCCCTCTG -3’ |
| *FT* | forward: 5’-TTGTTGGAGACGTTCTTGATCCGTTTAATAG -3’  reverse: 5’-CCTCCGCAGCCACTCTCCCTCTGACAATTG -3’ |
| *SOC1* | forward: 5’-GGGATCTCATGAAAGCGAAGTTTG-3’  reverse: 5’-CTTGAAGAACAAGGTAACCCAATGAA-3’ |
